# Droperidol in the Management of Phantom Limb Pain: Case Report

**DOI:** 10.5811/cpcem.1405

**Published:** 2023-05-26

**Authors:** Madison C. Winstead, Kimberly J. Wells, Gavin T. Howington

**Affiliations:** * University of Kentucky College of Pharmacy, Lexington, Kentucky; † University of Kentucky HealthCare, Department of Emergency Medicine, Lexington, Kentucky; ‡ University of Kentucky College of Pharmacy, Department of Pharmacy Practice and Science, Lexington, Kentucky; § University of Kentucky HealthCare, Department of Pharmacy Services, Lexington, Kentucky

**Keywords:** phantom limb pain, droperidol, case report, neuropathic pain, psychogenic

## Abstract

**Introduction:**

Phantom limb pain (PLP) is a poorly understood phenomenon experienced by amputees. The pain is typically classified as neuropathic, and there is no established first-line therapy. Droperidol is an antipsychotic with a wide array of pharmacologic activity including gamma-aminobutyric acid-A channel modulation, μ opioid receptor potentiation, dopamine-2-receptor blockade, and alpha-2-receptor agonism. Due to this broad therapeutic activity, droperidol is used for many off-label indications.

**Case Report:**

Our patient was a 25-year-old male with a history of lower limb amputation who presented for evaluation and management of an acute exacerbation of PLP. On arrival, the patient was in 10/10 pain (numeric pain rating scale) described as cramping and burning. He had been previously successfully managed with subdissociative ketamine. However, during a recent exacerbation he experienced an emergence reaction to ketamine. Literature guiding pharmacotherapy in the management of PLP is sparse and of low quality. Based on the prior emergence reaction to subdissociative ketamine we explored other pharmacotherapy options. Droperidol has a wide array of pharmacologic activity and is used off label for the management of some pain syndromes. Therefore, we administered an intravenous dose of droperidol 5 milligrams. Approximately 15 minutes after receiving droperidol the patient’s pain was visibly improved, and 30 minutes later he rated his pain at 3/10.

**Conclusion:**

The success in treating this patient provides encouragement for future research and bolsters confidence that droperidol could be another tool in the management of complex pain syndromes.

## INTRODUCTION

Phantom limb pain (PLP) is a phenomenon experienced by patients with both upper and lower limb amputations and is estimated to occur in 50–80% of amputees.^1^ These patients experience pain in a region of the body that is no longer present. Phantom limb pain is categorized as a neuropathic pain syndrome due to the description patients provide: tingling; throbbing; and pins and needles.^2^ Therefore, pharmacologic management is often extrapolated from other neuropathic pain syndromes.^1^ Many theoretical mechanisms have been postulated to explain PLP; one such theory is that PLP is psychogenic in nature as stress, anxiety, and depression are proposed to exacerbate PLP.^3^ Yet there are no established first-line pharmacologic treatments for this phenomenon. Based on current literature, a multimodal approach has the most success.^3^

Droperidol is a first-generation antipsychotic belonging to the butyrophenone class. Droperidol has similar dopaminergic blockade (D2-receptor antagonism) as haloperidol. However, droperidol also functions as an alpha-adrenergic blocker, alpha-2-receptor agonist, 5-hydroxytryptamine-3-receptor antagonist, histamine-1-receptor antagonist, gamma-aminobutyric acid (GABA)-A receptor modulator, sodium channel blocker, and μ opiate receptor potentiator.^4^ Droperidol has a US Food and Drug Administration (FDA)-approved indication for the management of postoperative nausea and vomiting.^5^ However, it is used off label for many indications including acute undifferentiated agitation, migraine, vertigo, acute on chronic abdominal pain, and refractory nausea and vomiting.^6^ Droperidol pharmacokinetics are as follows: onset of action is 3–10 minutes; peak effect is around 30 minutes; duration of action is 2–4 hours; absorption is rapid when administered intramuscularly; and half-life is approximately 1.7 hours for children and two hours for adults.^6^ Doses less than 10 milligrams (mg) typically are associated with few adverse effects.^7^

In 2001 the FDA mandated the inclusion of a boxed warning to droperidol concerning the risk of cardiovascular complications primarily associated with QT-prolonging effects and association with torsades de pointes.^8^ Utilization of droperidol then significantly decreased in the US, and many hospitals removed it from their formulary. Since that time a thorough review of the reports leading to the boxed warning has demonstrated many duplicate cases and harm associated more specifically with excessively high doses of droperidol (50 mg or greater).^8^ Over the past two decades new research has been published supporting the safety and efficacy of droperidol for many indications, and pharmaceutical manufacturer American Regent, Inc (Shirley, NY) has begun producing droperidol once again. Thus, there has been a resurgence in its use.

To our knowledge, no previous literature has described the use of droperidol for management of PLP. Phantom limb pain is a particularly challenging disorder to treat; therefore, we felt compelled to share our experience. We hope that the successful outcome in our patient might provide the impetus for further research into the use of droperidol for the management of PLP.

## CASE REPORT

A 25-year-old male presented to the emergency department (ED) for evaluation due to PLP. The patient was noted in the room to be visibly uncomfortable and in excruciating pain (numeric pain rating scale: 10/10.) He described his pain as cramping and burning in nature. This patient had a prior history of PLP consistent with his presentation as corroborated by a significant other at bedside and prior documented ED visits. Past medical history included above the knee amputation secondary to a high-speed motorcycle crash occurring approximately 1.5 years prior. The patient typically managed his chronic pain at home with gabapentin but would occasionally run out of his medication, provoking an exacerbation of PLP symptoms.

*CPC-EM Capsule*

What do we already know about this clinical entity?

*Phantom limb pain (PLP) is poorly understood and difficul to treat. Pain is typically classified as neuropathic, and there is no established first-line therapy*.

What makes this presentation of disease reportable?

*We successfully managed this acute exacerbation of PLP with droperidol. Medical literature has not previously reported this use of droperidol*.

What is the major learning point?

*Complex pain syndromes are difficult to manage. Leveraging what is known about the pharmacology of uncommonly used medications can lead to treatment success*.

How might this improve emergency medicine practice?

*Adding droperidol to the ED armamentarium of therapies for patients experiencing acute exacerbations of PLP can improve pain control*.

The patient was evaluated for an acute episode of PLP the day prior in a different ED within the same health system. At that visit, he was noted to be afebrile and hemodynamically stable. Vitals during the initial ED visit were as follows: blood pressure 104/57 millimeters of mercury (mm Hg), heart rate 118 beats per minute, temperature 36.9°Celsius (C), respiratory rate 22 breaths per minute, and his oxygen saturation was 94% on room air. The patient reported a 10 /10 on a numeric pain rating scale (worst pain ever). The patient affirmed that ketamine had worked in the past for breakthrough symptoms of PLP. Therefore, subdissociative ketamine (0.3 mg per kilogram) was ordered. Approximately four minutes after administering subdissociative ketamine, the patient experienced confusion, disorientation, and hallucinations, seemingly consistent with emergence phenomenon.^10^ This represented the first occurrence of emergence phenomenon for the patient, and it spontaneously resolved with no intervention provided. Additional pain-relieving medication provided during this hospital stay included intravenous (IV hydromorphone. Upon reassessment, the patient affirmed that his pain had resolved, and he felt comfortable going home.

Approximately 24 hours later, the patient presented to our ED for another acute exacerbation of PLP. He was again noted to be in visible discomfort and reported 10/10 pain. His vitals upon arrival were as follows: blood pressure 127/78 mm Hg; heart rate 118 beats per minute; temperature 36.7°C; respiratory rate 16 breaths per minute, and his oxygen saturation was 97% on room air. The ED pharmacist was consulted for analgesic recommendations due to the patient’s recent emergence reaction to ketamine. The pharmacist either subdissociative ketamine—with a plan to treat emergence if it occurred—or droperidol. The patient was presented with each option but was hesitant to receive subdissociative ketamine based on his prior emergence reaction. Droperidol was suggested due to its wide array of pharmacologic activity, safety profile, and the fact that most pain has some degree of psychogenic component.

Pharmacy recommended a dose of droperidol 5 mg administered via IV. Approximately 15 minutes after receiving droperidol, the patient’s pain had visibly improved, and he reported a 5/10 on the numeric pain rating scale. Thirty minutes later his pain continued to improve, noting a numeric pain rating scale score of 3/10. An hour later, the patient was in no acute distress, his pain was relieved, and he was ready to be discharged home from the ED. The patient was notably satisfied with the efficacy, safety, and onset of pain relief. Additionally, he has not presented again to our healthcare system for management of an acute exacerbation of PLP. However, it is difficult to appreciate whether this was due to long-term effects of droperidol or other factors as this case represents a single success.

## DISCUSSION

The medical literature has not previously reported the use of droperidol for PLP. We opted to use this therapy because PLP is considered neuropathic in nature. Droperidol antagonizes dopamine, serotonin, and histamine receptors, inhibits sodium channels, and potentiates GABA-A receptors. This novel case makes an argument for the use of droperidol for PLP when other medications have been trialed with failures or adverse reactions.

A Cochrane review published in 2011 describes the presentation, pathophysiology, and management of PLP. Included studies were both randomized or quasi-randomized in design and evaluated a variety of pharmacologic agents vs placebo, differing classes of pharmacologic agents, or no therapy. The review incorporates many studies detailing pharmacologic and non-pharmacologic management of PLP and discusses current uncertainty in the optimal treatment modality.^9^ Pharmacologic therapies currently described for the management of PLP include botulinum toxin A, opioids, N-methyl-D-aspartate (NMDA) receptor antagonists (ketamine, memantine, dextromethorphan), anticonvulsants, antidepressants, calcitonin, and local anesthetics. These medications can be used to help improve pain, function, mood, sleep, and quality of life. A summary of agents from the Cochrane review along with their proposed mechanism of action and evidence for usage is described below (Table).

In our patient’s case, opioids and a NMDA receptor antagonist had been previously trialed in the management of PLP. However, due to a lack of response and the development of an adverse event, alternative pharmacologic therapies were explored. Droperidol demonstrates its therapeutic effects by modulating serotonin, dopamine, histamine, and alpha-2 receptors. Therefore, it is not unreasonable to associate potential effectiveness of droperidol with improvement in PLP. For our patient, ketamine had worked in the past, but due to the development of emergence he requested another agent. Mechanisms of droperidol that are helpful in the analgesic effect are muscarinic antagonism, sodium channel blockade, and opiate receptor potentiation.^4^ This combination of psychogenic effects and analgesic effects make it a promising pharmacologic therapy in the management of PLP.

## CONCLUSION

We present a case that illustrates a potential unique indication for droperidol. Phantom limb pain can be difficult to manage, and the literature guiding pharmacologic therapy is sparse and of low quality. Droperidol was selected based on its broad array of pharmacologic activity and the underlying pathophysiology of PLP. The success in treating PLP in this patient gives encouragement for future research and bolsters confidence that droperidol could be another tool in the management of complex pain syndromes.

## Figures and Tables

**Table:** Evidence summary for management of phantom limb pain from 2016 Cochrane review.^9^

Drug/drug class	Proposed mechanism	Evidence
Botulinum Toxin A^8^	Prevents release of acetylcholine therefore blocking activation of the neuromuscular junction	Did not lower pain intensity assessed monthly for six months
Opioids^8^	Decrease cortical reorganization, block presynaptic nerve terminals and postsynaptic neurons involved in pain transmission	Oral and intravenous forms of morphine significantly reduced intensity of pain
NMDA receptor antagonists^8^	Blocks NMDA receptors on dorsal horn potentially decreasing pain manifestations	Equivocal results
Anticonvulsants^8^	Binds presynaptically to modulate release of excitatory neurotransmitters	Contradictory results
Antidepressants^8^	Inhibit presynaptic reuptake of serotonin and norepinephrine	Amitriptyline has been considered first-line for neuropathic pain, but a recent meta-analysis demonstrated a lack of good-quality studies and benefit; possibly only useful in select patients
Calcitonin^8^	Direct central action to inhibit neuronal firing in response to peripheral stimulation	Contrasting results
Local anesthetics^8^	Decrease spontaneous hyperactivity	Variable results

*NMDA*, N-methyl-D-aspartate.
